# Effects of *Croton rhamnifolioides* Essential Oil on *Aedes aegypti* Oviposition, Larval Toxicity and Trypsin Activity

**DOI:** 10.3390/molecules191016573

**Published:** 2014-10-14

**Authors:** Geanne K. N. Santos, Kamilla A. Dutra, Camila S. Lira, Bheatriz N. Lima, Thiago H. Napoleão, Patrícia M. G. Paiva, Claudia A. Maranhão, Sofia S. F. Brandão, Daniela M. A. F. Navarro

**Affiliations:** 1 Laboratório de Ecologia Química, Departamento de Química Fundamental, Universidade Federal de Pernambuco, 50670-901 Recife, PE, Brazil; 2 Laboratório de Glicoproteínas, Departamento de Bioquímica, Universidade Federal de Pernambuco, 50670-420 Recife, PE, Brazil; 3 Laboratório de Pesquisa e Desenvolvimento de Produtos Tecnológicos, Instituto Federal de Educação, Ciência e Tecnologia de Pernambuco, 50740-540 Recife, PE, Brazil

**Keywords:** *Aedes aegypti*, *Croton rhamnifolioides*, essential oil, storage, oviposition deterrent

## Abstract

Although numerous reports are available concerning the larvicidal potential of essential oils, very few investigations have focused on their mechanisms of action. In the present study, we have investigated the chemical composition of the leaf oil of *Croton rhamnifolioides* during storage and its effects on oviposition and survival of larvae of the dengue fever mosquito *Aedes aegypti*. In addition, we have established a possible mechanism of action for the larvicidal activity of the essential oil. GC-MS analyses revealed marked differences in the composition of oil that had been freshly isolated and that of a sample that had been stored in a sealed amber-glass vial under refrigeration for three years. However, both fresh and stored oil exhibited substantial larvicidal activities with LC_50_ values of 122.35 and 89.03 ppm, respectively, and oviposition deterrent effects against gravid females at concentrations of 50 and 100 µg·mL^−1^. These results demonstrate that the larvicidal effect of the essential oil was unchanged during three years of storage even though its chemical composition altered. Hence, the essential oil could be used in the preparation of commercial products. In addition, we observed that the trypsin-like activity of mosquito larvae was inhibited *in vitro* by the essential oil of *C. rhamnifolioides*, suggesting that the larvicidal effect may be associated with inhibition of this enzyme.

## 1. Introduction

*Croton* is a major genus of the family Euphorbiaceae and comprises some 1200 species of trees, shrubs and herbs that are widely distributed in tropical regions [1]. Members of the genus have been used in traditional medicine to treat a wide range of disorders, including malaria, inflammation, diabetes and cancer [2]. The biological properties of various *Croton* species have been investigated in laboratory studies involving murine models [3,4,5,6,7,8,9,10]. The chemical composition of *Croton* is somewhat diverse, although alkaloids [11,12], diterpenoids [13,14] and volatile oils comprising mono- and sesquiterpenoids [15,16,17,18], have been commonly reported.

In Brazil, some 356 species of *Croton* have been described [1] and these are distributed throughout the country in all biomes ranging from the tropical Amazon and Atlantic forests to the semi-arid northeastern regions [19]. *Croton rhamnifolioides* Pax & K. Hoffm. has been identified in several areas of the Brazilian Caatinga [20], a biome that is characterized by high temperatures with long and irregular periods of drought. This xerophytic shrub is used in folk medicine to treat stomach disorders, vomiting, *hemorrhagic* diarrhea and fever [21]. The pleasant aroma of the species is reportedly due to the presence of terpenoids in all parts of the plant [22], while the leaves are rich in flavonoids, and the roots and bark contain predominantly alkaloids, triterpenoids and steroids. However, studies regarding the biological activity of *C. rhamnifolioides* are scarce.

In recent years, dengue fever and dengue hemorrhagic fever have become a public health concern in a number of countries, especially in South America and Asia. According to the World Health Organization [23], approximately 2.5 billion people live in countries that are endemic for dengue and some 50-million dengue infections occur every year. In the absence of effective prophylactics or vaccines against the disease, the prevention of dengue fever is restricted to controlling the principal vector *Aedes (Stegomyia) aegypti* (Linneus, 1762).

Management of the mosquito typically involves the use of synthetic organic insecticides, however, continuous application of these compounds has led to the development of resistant populations of *A. aegypti* [24,25,26,27,28,29]. For this reason, considerable research has focused on the use of plant natural products in the control of various mosquito vectors including *A. aegypti* [30,31,32,33,34,35]. Although essential oils are present in different parts of the plant, there is particular interest in their extraction from aerial parts because of the possibility of sustainable production. The larvicidal effects of essential oils from several plant species have been studied. For example, Navarro *et al.* [36] demonstrated that the leaf oils obtained from members of the families Lauraceae and Piperaceae exhibited strong larvicidal activities (LC_50_ below 20 and 50 ppm, respectively). It has been accepted in the literature that an essential oil is active against *A. aegypti* larvae when LC_50_ < 100 ppm and strongly active when LC_50_ < 50 ppm.

The present paper aimed to (i) determine the chemical composition of the essential oil from leaves of *C. rhamnifolioides* and (ii) assess the influence of storage on the biological activities of the *C. rhamnifolioides* essential oil against *A. aegypti*. The potential use of the leaf oil from *C. rhamnifolioides* in controlling the spread of *A. aegypti* is described for the first time together with a possible mechanism of action.

## 2. Results and Discussion

The yield of essential oil obtained by hydrodistillation of dried leaves of *C. rhamnifolioides* was 0.80% (w/w), a value that is comparable with those reported previously for other species of *Croton* found in northeastern Brazil. For example, hydrodistillation of dried leaves of *C. heliotropiifolius* and *C. pulegiodorus* produced 0.2% and 5% of oil, respectively [37], while fresh branches of *C. adamantinus* gave 0.6% of oil [10] and fresh leaves and branches of *C. campestris* yielded 0.04% and 0.02% of oil, respectively [18]. Additionally, Camurça-Vasconcelos *et al.* [38] reported a 3.15% yield of essential oil following steam distillation of the dried aerial parts of *C. zehntneri*.

A total of 57 compounds, mainly mono- and sesquiterpenoids, were identified by GC-MS analysis of the freshly isolated essential oil of *C. rhamnifolioides* (Table 1), and these components represented more than 92% of the total oil. The major constituent of the oil was the oxygenated sesquiterpene sesquicineole (16.79%), followed by the monoterpene α-phellandrene (12.83%), the oxygenated monoterpene 1,8-cineole (7.24%), and the sesquiterpene (*E*)-caryophyllene (6.33%). The characteristic features of the essential oil of *C. rhamnifolioides* obtained in the present study resembled those reported for various species of *Croton*, including *C. heliotropiifolius* and *C. pulegiodorus* [37], *C. argyrophylloides* and *C. sonderianus* [39], *C. nepetaefolius* [40], *C. adamantinus* [10], and *C. campestris* [18], but differed from those of *C. zehntneri*, *C. nepetaefolius* [39] and *C. regelianus* [41], the major components of which were *trans*-anethole, methyl eugenol and ascaridole, respectively.

The chemical composition of an essential oil can change after extraction depending on the storage conditions applied. Misharina and co-workers [42] reported that storage of the essential oil of marjoram in the dark for one year resulted in insignificant alterations in composition, while considerable changes were detected in samples of the same oil that had been exposed to light during storage. In the present study, the essential oil of *C. rhamnifolioides* was stored in a sealed amber-glass vial maintained at −5 °C for three years, conditions that have previously been shown to produce the smallest changes in the quality of volatile oil samples [43]. GC-MS analysis of the composition of the stored essential oil revealed that the relative percentages of 1,8-cineole (18.61%) and *o*-cymene (14.64%) had increased during storage, while that of sesquicineole (1.77%) had decreased. In contrast, the relative concentration of α-phellandrene was similar in both fresh and stored oil samples, and this prompted us to assess the larvicidal activity of this monoterpene hydrocarbon.

**Scheme 1:**
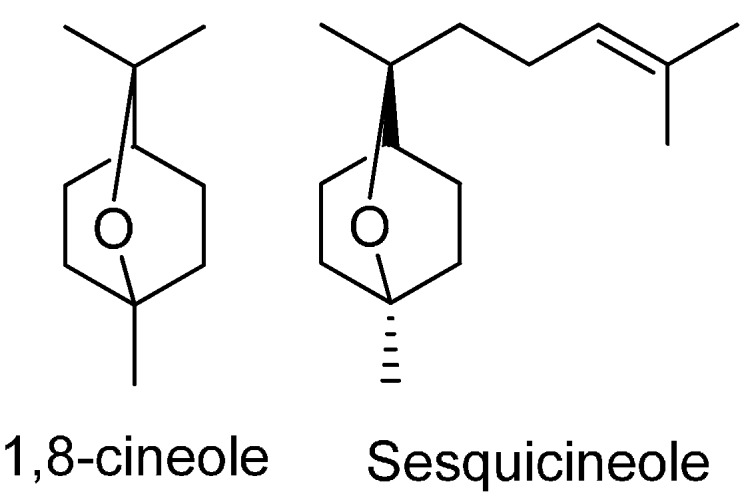
Chemical structures of 1,8-cineole and sesquicineole.

The observed increases in the relative percentages of oxygenated monoterpenes in the stored oil are in agreement with the findings of Turek *et al.* [44]. These authors also reported alterations in the amounts of *p*-cymene in essential oils submitted to different storage conditions, which were similar to those observed for *o*-cymene in the present study. Interestingly, 1,8-cineole and sesquicineole (Scheme 1) have similar biosynthetic pathways [45,46] and so the increase in the amount of 1,8-cineole requires careful investigation in future work.

**Table 1:** Volatile compounds identified in fresh and stored samples of essential oil from *Croton rhamnifolioides.*

Component ^a^	Fresh Oil [%]	LRI calc. ^b^	Stored Oil [%]		LRI calc. ^b^	LRI Lit. ^c^
Tricyclene	0.16	921	0.52		920	920
α-Thujene	1.49	924	2.17		926	926
α-Pinene	4.74	932	9.52		932	932
Camphene	0.65	946	2.4		946	946
Sabinene	3.04	969	7.18		972	972
β-Pinene	0.55	974	0.69		974	974
Myrcene	0.36	988	0.68		991	991
**α-Phellandrene**	**12.83**	1002	**8.37**		1003	1003
α-Terpinene	0.31	1014	-		-	1016
*o-*Cymene	4.60	1022	14.64		1023	1023
Sylvestrene	3.62	1025	3.99		1028	1027
**1,8-Cineole**	**7.24**	1026	**18.61**		1030	1030
Benzyl alcohol	0.20	1026	-		-	1033
(*E*)-β Ocimene	0.75	1044	0.34		1049	1048
γ-Terpinene	1.09	1054	-		-	1058
Terpinolene	0.24	1086	-		-	1088
Linalool	1.32	1095	1.27		1100	1100
Dehydro-sabina ketone	0.16	1117	-		-	1120
(*Z*)-β-Terpineol	0.08	1140	-		-	1139
Camphor	0.06	1141	0.45		1144	1144
Pinocarvone	0.02	1160	-		-	1162
Borneol	0.83	1165	-		-	1165
Terpinen-4-ol	1.90	1174	0.48		1177	1177
α-Terpineol	1.91	1186	0.7		1190	1190
Myrtenol	0.31	1194	-		-	1196
Methyl chavicol	0.06	1195	-		-	1198
Thymol methyl ether	0.03	1232	-		-	1235
Bornyl acetate	0.33	1284	0.72		1287	1286
Thymol	0.05	1289	-		-	1292
Carvacrol	0.20	1298	-		-	1301
Myrtenyl acetate	0.02	1324	-		-	1326
δ-Elemene	0.03	1335	-		-	1338
α-Copaene	0.19	1374	0.43		1378	1377
β-Bourbonene	0.07	1387	-		-	1386
β-Elemene	0.35	1389	1.36		1394	1393
(*Z*)-α-Bergamotene	0.05	1411	-		-	1417
(*E*)-Caryophyllene	6.33	1417	4.37		1422	1422
(*E*)α-Bergamotene	0.14	1432	-		-	1438
(*Z*)-β-Farnesene	0.04	1440	-		-	1445
α-Humulene	1.17	1452		0.96	1456	1457
9-*epi*-(*E*)-Caryophyllene	0.80	1464		0.95	1464	1465
γ-Muurolene	0.04	1478		-	-	1481
Germacrene D	0.99	1484		-	-	1485
Viridiflorene	0.14	1496		0.71	1489	1490
Bicyclogermacrene	4.59	1500		2.44	1499	1501
(*Z*)-β-Guaiene	0.10	1502		-	-	1504
α-Bulnesene	0.05	1509		-	-	1506
Germacrene A	0.21	1508		0.84	1508	1510
**Sesquicineole**	**16.79**	1515		**1.77**	1516	1518
δ-Cadinene	0.45	1522		0.44	1526	1527
Germacrene B	0.06	1559		-	-	1556
Spathulenol	4.14	1577		4.98	1580	1580
Caryophyllene oxide	3.22	1582		2.89	1586	1586
Viridiflorol	0.09	1592		-	-	1596
*epi*-α-Cadinol	2.33	1638		3.16	1644	1643
α-Bisabolol	0.72	1685		-	-	1685
Eudesma-4(15),7-dien-1β-ol	0.23	1687		-	-	1688
Monoterpene hydrocarbons	32.94			48.33		
Oxygenated monoterpenes	15.85			24.4		
Sesquiterpene hydrocarbons	15.75			12.5		
Oxygenated sesquiterpenes	27.52			12.8		
Total	92.47				98.03	

^a^ Constituents listed in order of elution from a non-polar DB-5 capillary column; ^b^ Linear retention indices calculated from retention times in relation to those of a series of *n-*alkanes separated on a non-polar DB-5 capillary column; ^c^ Linear retention indices from the literature.

With the aim of developing novel methods for the control of *A. aegypti* populations, the larvicidal activities of samples of essential oils obtained from dried leaves of *C. rhamnifolioides* were evaluated. Both fresh and stored samples of the essential oil exhibited substantial larvicidal activity against 4th instar *A. aegypti* (LC_50_ = 122.3 ± 3.7 and 89.03 ± 1.94 µg·mL^−1^, respectively; Table 2). The larvicidal properties of sesquicineole, one of the four major components of the fresh oil, could not be assayed directly because the standard compound was not available commercially. However, since storage of the oil was associated with an increase in larvicidal activity but to a decrease in the amount of sesquicineole present, it is reasonable to assume that this constituent made little or no significant contribution to the overall larvicidal activity of the oil. On the other hand, bioassay of standard α-phellandrene revealed strong activity against *A. aegypti* larvae with an LC_50_ value of 39.3 ± 1.0 µg·mL^‑1^, while standard 1,8 cineole was not effective in killing larvae at concentrations below 150 µg·mL^−1^. According to the literature, the contributions to larvicidal activity of 1,8-cineole (LC_50_ in the range 74.9–1381 µg·mL^−1^), α-pinene (LC_50_ > 300 µg·mL^−1^) and (*E*)-caryophyllene (LC_50_ in the range 88.3–1202 µg·mL^−1^) vary from moderate to low [36,37,38,39,40,41,42,43,44,45,46,47]. Moreover, our data relating to the larvicidal effect of α-phellandrene are in agreement with the results published by Perumalsamy *et al.* [48] and Cheng *et al.* [49]. However, it is also possible that *o*-cymene might contribute to maintaining the larvicidal activity in stored samples of *C. rhamnifolioides* oil since, although larvicidal data for standard *o*-cymene are not available in the literature, the LC_50_ value for the isomer *p*-cymene is reportedly within the range 19.2 to 37.1 µg·mL^−1^ [47].

**Table 2:** Larvicidal activities of fresh and stored samples of essential oil of *Croton rhamnifolioides* and their major constituents.

Test Sample	LC_50_ ± SE [µg·mL^−1^]	Confidence Interval [µg·mL^−1^]	Χ^2^ Test	*p* Level
*C. rhamnifolioides* (fresh essential oil)	122.3 ± 3.7	115.1–129.6	0.2	1.00
*C. rhamnifolioides* (stored essential oil)	89.0 ± 1.9	85.2–92.8	0.6	0.90
α-Phellandrene	39.3 ± 1.0	37.3-41.3	1.4	0.77
1,8-Cineole	>100	-	-	-

Activities against *A. aegypti* larvae of essential oils derived from a number of *Croton* species have been reported in the literature. Thus, oils from *C. zehntneri*, *C. nepetaefolius, C. argyrophyloides* and *C. sonderianus* showed LC_50_ values of 28, 84, 102 and 104 µg·mL^−1^, respectively, against 3rd instar *A. aegypti* [40]. Additionally, all of these oils exhibited ovicidal and pupicidal effects against the dengue vector [39]. Dória *et al.* [37] assayed essential oils from *C. pulegiodorus* and *C. heliotropiifolius* against larvae of *A. aegypti* and reported LC_50_ values of 159 and 544 µg·mL^−1^, respectively. The essential oils from specimens of *C. regelianus* collected at two different locations in the Brazilian state of Ceará, were highly effective against 3rd instar *A. aegypti* (LC_50_ in the range 24.22 and 66.74 µg·mL^−1^), and this activity was apparently associated with the major oil component ascaridole [41].

**Figure 1:**
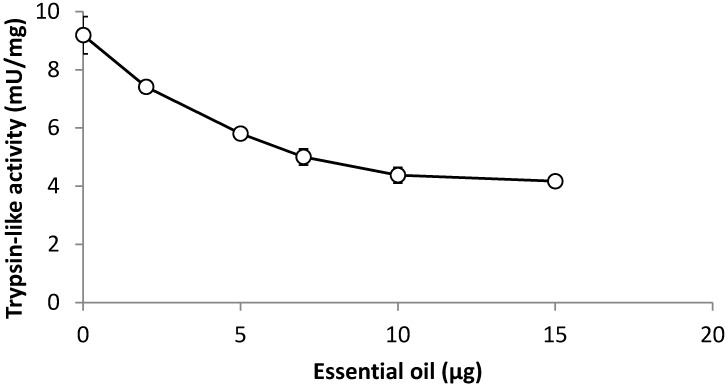
Effect of the leaf oil of *Croton rhamnifolioides* on the trypsin-like activity of *Aedes aegypti* 4th instar larvae.

Although there are various reports on the larvicidal activity of essential oils against *A. aegypti*, the mechanisms of action are not yet understood. In this study, we tested the hypothesis that the leaf oil of *C. rhamnifolioides* may interfere with the trypsin-like activity of the larval gut. The results revealed that the leaf oil inhibited trypsin-like activity of 4th instar *A. aegypti* in a dose-dependent manner (Figure 1), and this inhibitory effect may be associated with larvicidal activity. Trypsin is a serine protease that occurs widely in insect guts and the impairment of its activity may result in poor nutrient absorption and non-availability of essential amino acids. Trypsin-like enzymes from *A. aegypti* larvae have been reported as targets for other plant-derived larvicides as exemplified by the trypsin inhibitor found in *Moringa oleifera* flowers [50] and the lectin detected in *Myracrodruon urundeuva* leaves [51].

Other potential targets of essential oils are TRP-type ion channels, acetylcholinesterase and receptors of tyramine, octopamine and GABA [52]. Kostyukovsky *et al.* [53] found that essential oils from Israeli plants *were able to* activate octopaminergic receptors present in the abdominal segments of *Helicoverpa armigera*, *while* Enan [54] suggested that the toxicity of cinnamic alcohol, eugenol, *trans*-anethole, and 2-phenethyl propionate against *Drosophila melanogaster* is mediated by octopamine receptors. A molecular docking study conducted by Khanikor *et al.* [55] indicated that the terpenes carvacrol, eucalyptol and eugenol act as acetylcholinesterase inhibitors and bind to the octopamine receptors of *A. aegypti*. However, Anderson and Coats [56] demonstrated that the terpenoids carvacrol and nootkatone did not inhibit acetylcholinesterase in *A. aegypti*. Our study opens new windows on the mechanisms of action of essential oils against *A. aegypti* by reporting the inhibitory effect of *C. rhamnifolioides* oil on a digestive enzyme from larvae of this species.

**Figure 2:**
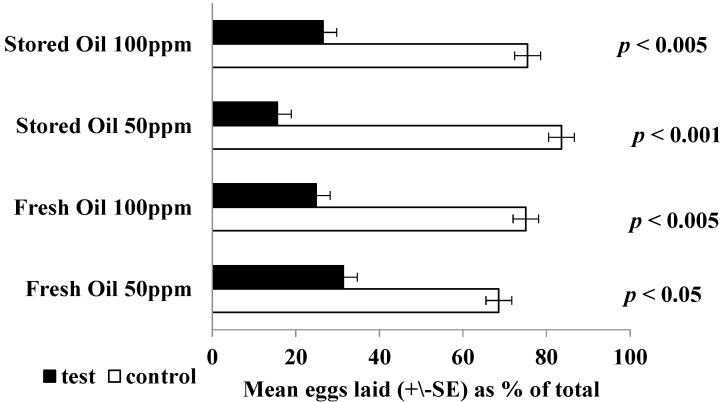
Oviposition responses of gravid *Aedes aegypti* to aqueous solutions of fresh and stored samples of essential oil from *Croton rhamnifolioides*. The values represent mean percentages (±SE) of the total eggs laid after 16 h in response to the treatment. Each assay involved ten mosquitoes and was replicated eight times.

The selection by gravid female mosquitoes of suitable sites for oviposition is guided by various factors including visual and olfactory cues. The presence of an oviposition deterrent in the water can result in the laying of few, if any, eggs at that site [57]. In the present study, aliquots of fresh and stored essential oils from *C. rhamnifolioides* exhibited an oviposition deterrent effect at 50 and 100 µg·mL^−1 ^with significantly smaller numbers of eggs (<50%) being laid in vessels containing oil solutions compared with those containing a control solution (Figure 2).

It is reported that essential oils from a wide variety of species, including *C. zehntneri*, *C. argyrophylloides* [39], *Piper marginatum* [58] and *Cananga odorata* [59], exhibit oviposition deterrent properties similar to those reported in the present study.

## 3. Experimental Section

### 3.1. Plant Material

Specimens of *C. rhamnifolioides* were collected at a private farm located in the municipality of Serra Talhada, PE, Brazil, in October 2009. Plant material was identified by Dr. Elba Maria Nogueira Ferraz Ramos (Instituto Federal de Educação, Ciência e Tecnologia de Pernambuco, Recife, PE, Brazil), and a voucher specimen has been deposited at the Herbarium Vasconcelos Sobrinho, Universidade Federal Rural de Pernambuco, Recife, PE, Brazil, with voucher number 49,855.

### 3.2. Extraction of Essential Oil

Leaves of *C. rhamnifolioides* were separated manually and left to dry naturally under shade conditions for three days. Dried leaves (100 g) were submitted to hydrodistillation with 1 L of distilled water for 2 h in a Clevenger-type apparatus, following which the essential oil layer was separated, dried over anhydrous sodium sulfate and transferred to amber-glass vials. The yield of fresh essential oil was determined as the quotient of the weight of oil collected and the dry weight of plant material extracted. The chemical composition and biological activity of the oil was measured soon after extraction (fresh oil) and after storage in a hermetically sealed amber-glass vial for three years at −5 °C (stored oil).

### 3.3. GC-MS Analyses

Analyses of fresh and stored samples of essential oils were carried out using an Agilent Technologies (Palo Alto, CA, USA) 5975C single quadrupole GC-MS equipped with a J & WScientific non-polar DB-5 fused silica capillary column (30 m × 0.25 mm i.d.; film thickness 0.25 mm). The oven was held initially at 60 °C for 3 min, then increased at 2.5 °C·min^−1^ to 240 °C, and finally held at this temperature for 10 min. The carrier gas was helium supplied at a constant pressure of 100 kPa, and the split/splitless injector was maintained at 250 °C. The applied ionization potential was 70 eV, the scan range was from 40 to 350 *m*/*z* and the scan rate was 0.5 scans·s^−1^. Samples (1 µL containing 2 mg·mL^−1^ of essential oil in hexane) were introduced onto the column with the injector in the splitless mode. Linear retention indices (LRI) were determined for the individual components of the essential oil by co-injection of a sample with a mixture of C_8_–C_30_ linear hydrocarbons under the conditions described above, and subsequent application of the Van den Dool and Kratz [60] equation. Constituents were initially identified by comparison of LRI values with those published in the literature, and the identities confirmed by matching acquired MS with those stored in the library of the GC-MS system (NIST, Gaithersburg, MD, USA and Wiley, New Jersey, NJ, USA) and with other published data [61].

### 3.4. Mosquito Population

A population of *A. aegypti* (Rockefeller strain) was maintained in the laboratory at 28 ± 1 °C and 70% ± 5% relative humidity under a 14 h photoperiod. Adult mosquitoes were reared in wooden cages (33 × 33 × 33 cm) and maintained on 10% sucrose solution, while females were blood-fed on pigeons once a week. Eggs were collected three days after the blood meal by placing a recipient, containing tap water and a piece of filter paper to provide support for oviposition, inside the cage. Eggs were hatched by submersion in tap water, and larvae were reared in plastic basins and fed on a diet of commercial cat food (Whiskas^®^, Mars Petcare Corporate, Guararema, Brazil).

### 3.5. Larvicidal Bioassays

Bioassays were conducted soon after extraction of the essential oil (fresh oil) and after three years of storage at −5 °C (stored oil). In both cases, a stock colloidal solution (containing 200 µg·mL^−1^) was prepared by dissolving 20.00 mg of oil sample in 1.4 mL of ethanol and completing to 100 mL with distilled water to produce a homogenous solution. Larvicidal activities were evaluated using the method recommended by the World Health Organization [23] as modified by Navarro *et al.* [62]. Early 4th instar larvae of *A. aegypti* (recognized by the lighter color of the head and pronota) were transferred to disposable cups (20 larvae per cup) containing essential oil at different concentrations prepared by dilution of the stock solution with distilled water. Five concentrations of oil solution (80, 100, 120, 140 and 160 µg·mL^−1^ for fresh oil; 75, 80, 90, 100 and 120 µg·mL^−1 ^ for stored oil) were assayed in order to determine median lethal concentration (LC_50_) values, and four replicate assays were carried out for each sample concentration. For comparison purposes, bioassays were also performed with standard α-phellandrene (SAFC: purity 99%) at concentrations of 30, 35, 40, 45 and 50 µg·mL^−1^, and with standard 1,8 cineole (Sigma-Aldrich: purity 99%) at concentrations of 50, 100, 150 and 200 µg·mL^−1^. Larval mortalities, assessed as lack of response to stimulus or larvae not rising to the surface, were determined after 24 and 48 h, and LC_50_ values were calculated from 48 h mortality data by Probit analysis using StatPlus2008 software. Negative controls (distilled water containing the same amount of ethanol as the test sample) were included in each assay and the absence of larvae mortality was confirmed. An aqueous solution containing 1 µg·mL^−1^ of Temephos, a commonly used larvicide, formed the positive control and 100% larvae mortality was verified.

### 3.6. Gut Extracts from A. aegypti 4th Instar Larvae

Groups of fifty 4th instar larvae were collected and immobilized by cooling at 4 °C for 10 min. The gut of each larva was subsequently removed using a needle (8 mm long; 0.3 mm diameter), transferred to a 2 mL tissue grinder and immediately homogenized with 1 mL of 0.1 M Tris-HCl (pH 8.0) containing 0.02 M CaCl_2_ and 0.15 M NaCl. The homogenate was centrifuged (9000 ×*g*; 4 °C, 15 min) and the supernatant (gut extract) was collected and evaluated for protein concentration [63] and trypsin-like activity.

### 3.7. Effect of Leaf Oil on Trypsin-Like Activity from Larvae

Trypsin-like activity was determined by incubating larval gut extract (15 µL) for 30 min at 37 °C with 8 mM *N*-benzoyl-dl-arginyl-ρ-nitroanilide (BApNA, 5 μL) in 0.1 M Tris-HCl pH 8.0 (180 μL). Hydrolysis of the substrate was followed by measurement of the absorbance at 405 nm using a microplate reader (μQuant, MQX200; BioTek Instruments, Inc., Winooski, VT, USA). One unit of trypsin activity was defined as the amount of enzyme that hydrolyzes 1 µmol of BApNA per min. Specific trypsin-like activity was defined as the ratio between enzyme activity and protein amount (mg) in the assay.

The effect of leaf oil on trypsin-like activity was determined according to Pontual *et al.* [50]. Larval gut extract (15 µL) was incubated with the oil sample (2.0 to 15.0 µg dissolved in dimethyl sulfoxide) for 10 min at 28 °C, following which the substrate (8 mM BApNA; 5 μL) was added and the mixture incubated for a 45 min at 37 °C prior to measurement of the absorbance at 405 nm. All assays were performed in triplicate along with the reaction blanks containing substrate or gut extract only.

### 3.8. Oviposition Bioassays

Solutions containing aliquots of fresh or stored essential oils at concentrations of 50 and 100 µg·mL^−1^ were prepared by dissolving the appropriate amount of oil in 1.4 mL of ethanol and completing to 200 mL with distilled water. Ten gravid 7 day old *A. aegypti* females were transferred to a cage (33 × 21 × 30 cm) containing two disposable cups, one filled with 25 mL of essential oil solution and the other with 25 mL of control solution (distilled water containing the same amount of ethanol as the oil sample). Filter paper was placed on the internal surface of each cup to provide a support for oviposition, and the two cups were placed at diagonally opposite corners of the cage. For each treatment, eight cages were prepared. The bioassay was conducted in the dark for 16 h at 28 ± 1 °C and 70% ± 5% relative humidity. The oviposition response was subsequently determined by counting the numbers of eggs laid on the filter papers. Mean values obtained in each of the treatments were compared using the Student *t*-test [58,62].

## 4. Conclusions

α-Phellandrene and the essential oil of *C. rhamnifolioides* could find application in the control of *A. aegypti,* since both inhibit the oviposition of females at the breeding sites and kill mosquito larvae before they become adults. The retention of the larvicidal and oviposition deterrent activities of the essential oil during storage demonstrates that *C. rhamnifolioides* leaf oil could be used in the long term to combat the spread of the dengue mosquito. The mechanism of action of the leaf oil may be associated with its ability to inhibit trypsin-like activity in 4th instar larvae of *A. aegypti.*
